# Air Pollution Monitoring Around Residential and Transportation Sector Locations in Lagos Mainland

**DOI:** 10.5696/2156-9614-8.19.180903

**Published:** 2018-08-21

**Authors:** Henry E. Obanya, Nnamdi H. Amaeze, Olusola Togunde, Adebayo A. Otitoloju

**Affiliations:** Ecotoxicology Laboratory, Department of Zoology, University of Lagos, Akoka-Yaba, Lagos, Nigeria

**Keywords:** urban air quality, fossil fuel, public health, vehicular exhaust, road traffic

## Abstract

**Background.:**

Industrialization and urbanization, while associated with increased productivity, are also potential causes of increased air pollution. Urban air quality has the potential to affect the health and wellbeing of residents of urban areas.

**Objectives.:**

The present study investigated the levels of air pollutants around residential areas and transport sector locations (TSLs) in Lagos, Nigeria. Residential areas were defined as areas around inner streets and living quarters, while TSLs included busy roads, dual carriage roads, bus stops and major car parks in the Yaba Local Council Development Area of Lagos Mainland, Lagos, Nigeria.

**Methods.:**

Air quality parameters were assessed *in situ* using calibrated hand-held devices at selected residential and TSLs. Each sampling location was geo-referenced and concentrations of the various parameters were used to plot distribution maps.

**Results.:**

The findings from the monitoring exercise showed that levels of the measured air pollutants: carbon monoxide (CO), particulate matter (PM_10_ and PM_2.5_), sulphur dioxide (SO_2_), noise, temperature and humidity were within the ranges of 1.00 – 6.0 5.97 ppm, 43.345.2 – 127.2159.7 μg/m^3^, 20.3 23.25 – 69.058.16 μg/m^3^, 0.0 0 – 0.20.17 ppm, 47.7 50 - 65 70.1 dB, 26.2227.2 – 35.536.7°C and 57.0157.6 – 91.8492.3%, respectively, around residential areas. Values of the measured air pollutants at the TSLs ranged as follows: 2.011.0 – 5.397.7 ppm, 103.3360.7 – 179.77404.0 μg/m^3^, 50.2832.3 – 91.01184.0 μg/m^3^, 0.00 – 0.40 ppm, 64.2153.1 – 71.1376.3 dB, 27.1826.2 –27.9332.6°C and 60.3660.0 – 75.0178.0%, respectively. Hydrogen sulphide (H_2_S), ammonia (NH_3_), nitrogen oxide (NO_2_) were below detection limits in both sampling locations while volatile organic carbons (VOCs) ranged from 0.00 – 0.10 ppm in the TSLs.

**Discussion.:**

Most assessed air quality parameters were significantly higher around bus stops (P < 0.05), except for CO and humidity. In addition, PM_10_ and PM_2.5_ were much higher than the World Health Organization (WHO) guidelines. The results indicated that the quality of air (particulate matter) in the study area was poor, especially in the TSLs.

**Conclusions.:**

The Federal Ministry of Environment, through its relevant agencies, must create policies to address urban air pollution, taking into consideration long term exposures and people that are most vulnerable within the population.

**Competing Interests.:**

The authors declare no competing financial interests.

## Introduction

Air pollution is the presence in the atmosphere of one or more contaminants in such quantity and for such duration as to be injurious, or tending to be injurious, to human health or welfare, animal or plant life.^1^ The World Health Organization (WHO) estimates that 10% of global mortality, amounting to 7 million people, resulted from air pollution in 2012.^2^ In the 2016 report, 2.9 million annual deaths were reported, of which more than 85% occurred in low- and middle-income countries.^3^ Rapid growth in motor vehicle traffic and rapid industrialization are contributing to high levels of urban air pollution. The major sources of air pollutants in the environment include emissions from vehicles, electricity generators and industries, road and building construction activities, as well as mining. Motor vehicle emissions constitute the main source of fine and ultrafine particles and have a serious impact on urban air quality and public health.^4^ Globally, the hazardous impact of air pollution on human health and the environment has been on the rise, particularly in developing countries where most people still generate their own electricity by means of fossil fuel (diesel and petrol) powered electricity generator sets for both commercial and domestic use.

Among the major air pollutants of concern are carbon monoxide, carbon dioxide, oxides of nitrogen, oxides of sulfur, particulate matter, noise and volatile organic compounds such as benzene, polycyclic hydrocarbons and formaldehyde.^5^,^6^ Each of these pollutants can have severe consequences in both the short- and long-term, resulting in acute and chronic toxicity effects. Many of these substances have harmful effects on bone marrow, the spleen and lymph nodes.^7^ The circulatory system is especially vulnerable to toxins in exhaust fumes and exposures have been linked to asphyxiation and anemia.^8^ Exposure to air pollutants has been associated with increased risk of upper respiratory tract diseases such as asthma, inflammation, fibrosis and chronic obstructive pulmonary disease, exacerbation of heart disease due to hypertension and degeneration of the cells which line blood vessels, irreparable damage to the central nervous system, as well as cancers.^9–13^

Combustion of fossil fuel in engines such as those which burn gasoline and diesel is the major cause of air pollution globally, releasing carbon dioxide and carbon monoxide into the air. Carbon monoxide emanates primarily from vehicular and generator exhausts and deprives the bloodstream of oxygen necessary for many vital functions within the body by competitively binding with hemoglobin.^14^ Carbon dioxide is also a major problem, leading to global warming as a result of its shielding greenhouse effect.^15^ However, global warming is a catchall phrase that encompasses a great number of problems. Increased global temperatures have been associated with a gradual reduction in the arctic sea ice caps and disruption of crop growing seasons.^15^,^16^ Unfortunately, this is just the beginning of a snowball effect that filters down through virtually every life form on the planet. Animal habitats are being destroyed at record speed, which causes them to seek alternative sources of food and shelter, further increasing extinction pressure. As a result, it is projected that some species could very well become extinct within our lifetime.^17^ In addition, due to a change in the length of growing seasons, many crops will be negatively affected, with the potential for escalating food prices. The melting polar caps will cause an increase in sea level which will most certainly submerge many islands and coastal areas, forcing both animals and humans farther inland.

**Abbreviations**
*FMEnv*: Federal Ministry of the Environment
*TSL*: Transport sector location
*USEPA*: United States Environmental Protection Agency
*WHO*: World Health Organization
*DLI*: Distance Learning Institute
*WAEC*: West African Examination Council
*S/N*: Serial number

According to Mannucci and Massimo, low- and middle-income countries have experienced an intense process of urbanization and industrial development in a very short period of time compared to most developed countries that have long completed industrialization programs.^18^ These low- and middle-income countries therefore have had the largest air pollution-related burdens in recent years. The WHO's latest records of urban air quality show that 98% of cities in low- and middle-income countries with more than 100,000 inhabitants do not meet its air quality guidelines, compared to 56% of cities in high-income countries. This means that globally, over 80% of people living in urban areas that monitor air pollution are exposed to air quality levels that exceed the WHO limits.^19^ Nigeria has some of the most polluted cities in the world based on available records, and three of the ten most polluted cities in the world are in Nigeria: Onitsha (PM_10_=594 μg/m^3^), Kaduna (PM_10_=423 μg/m^3^) and Aba (PM_10_ = 373 μg/m^3^).^20^ Lagos is Nigeria's commercial hub and is estimated to have a population of over 20 million.^21^ High population density and attendant road traffic coupled with electricity shortages causing high reliance on generators by households pose serious air quality challenges for urban residents.

The aim of the present study was to monitor air pollutants around household/transportation sector locations in Lagos mainland and to illustrate the distribution pattern using ArcGIS software. This will provide valuable data as a buildup to a future city-wide evaluation.

## Methods

**Figure 1:**
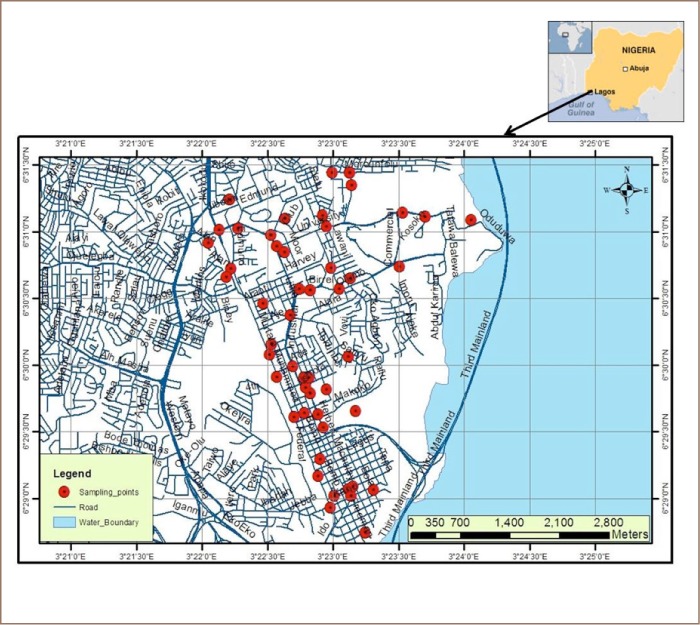
Study area showing sampling locations—transport sector locations and households (red)

The study was conducted around transport sector locations (TSLs) and residential areas of the Mainland Local Government Area, an administrative unit of Lagos State, Nigeria (*Figure 1*). It is a highly urbanized area characterized by colonial estates, modern streets and coastal slums. Most major roads are busy and traffic density is high, but the inner street and many residential quarters experience less traffic. However, the use of generators by households to produce domestic electricity, together with cooking, are major sources of air pollution around residential areas.

### Study design

The sampling was done at residential areas and TSLs in the study area and the average values for respective parameter readings were recorded at each sampling point using calibrated sampling devices. The assessed parameters included noise, carbon monoxide (CO), particulate matter (PM_10_ and PM_2.5_), sulphur dioxides (SO_2_), hydrogen sulphide (H_2_S), ammonia (NH_3_), nitrogen oxide (NO_2_), volatile organic carbon (VOCs), temperature and humidity. All measurements were done in one-hour triplicates (i.e. repeated three times- morning, afternoon and night) per day, except for CO which was performed for two-hour triplicates per day (and converted to a 1-hour mean) based on limits set by the United States Environmental Protection Agency (USEPA) and the Nigerian Federal Ministry of the Environment (FMEnv). WHO values, usually based on 24-hour measurements have also been included in Table 1 to provide a broader view of comparison.

**Table 1:** Air Quality Means in Households and Transport Sector Locations in Lagos Mainland

Parameters	Locations	Mean ± SD	USEPA^26^	WHO^27^	FMEnv Limit^28^
Carbon monoxide (ppm)	Transport Sector Locations	3.3 ± 1.7^a^	35	25	10
	Residential	3.1 ± 1.5^a^			
Sulphur dioxide (ppm)	Transport Sector Locations	0.14 ± 0.09^a^	75 ppb	20 (24hour mean)	-
	Residential	0.06 ± 0.07^b^			
Noise (dB(A))	Transport Sector Locations	67.7 ± 6.0^a^	-	70	85-90
	Residential	58.2 ± 6.8^b^		50-55	
Particulate Matter 10 (μg/m^3^)	Transport Sector Locations	144.1 ±76.6^a^	150	50 (24hour mean)	250
	Residential	73.4 ± 27.5^b^			
Particulate Matter 2.5 (μg/m^3^)	Transport Sector Locations	69.6 ± 35.1^a^	35	25 (24hour mean)	250
	Residential	34.9 ± 12.1^b^			
Volatile Organic Carbons (ppm)	Transport Sector Locations	0.023 ± 0.04^a^		-	-
	Residential	0.0 ± 0.0^b^			
Nitrogen oxide	Transport Sector Locations	0.0 ± 0.0^a^	53ppb	200	
	Residential	0.0 ± 0.0^a^			
Temperature (°C)	Transport Sector Locations	29.2 ± 2.0^a^		-	-
	Residential	33.3 ± 2.2^a^			
Humidity (%)	Transport Sector Locations	68.9 ± 6.8^a^		-	-
	Residential	67.4 ± 8.5^a^			

### Sampling

Sampling was performed between September and November 2017. A total of 52 samples were collected monthly over the course of the study (21 around households and 31 around TSLs).

Sampling was done *in situ* using various calibrated hand-held devices and each location was geo-referenced using a handheld GARMINTM 72H GPS device. The detector tube method was used to measure carbon monoxide levels (model: DSM8922).^22^ The exposure duration was set at 2 hours at a time per place. The average values for each locations were computed.^23^ Particulate matter levels (PM_10_ and PM_2.5_) were measured using a handheld air tester (CW – HAT 200), designed to measure the particulate level in the air for 60 seconds at a flow rate of 500 ml. The sensitivity of the unit ranged from 0 – 500 μg/m^3^. The handheld air tester, being a multi-air quality meter, was also used to measure humidity and temperature. Readings were obtained over a range of one hour, repeated three times, and the average was recorded.

Sulphur dioxide was measured using an ITX multi-gas monitor for one-hour exposure time.^24^ The monitor has a range of 99.9 ppm and a resolution of 0.1 ppm. Readings were obtained over a range of one hour, repeated three times, and then the average was recorded.

The levels of H_2_S, NH_3_ and NO_2_ were monitored using a GC310 gas detector; a flexible portable gas detector for multi-gases (up to 4 gases). The sensitivity of the units ranged from 0 – 100.0 ppm, 0–100 ppm and 0 – 20.0 ppm. Readings were obtained over a range of one hour and then the average was recorded.

A MultiRAE IR (PG M54) meter was used to assess VOCs. It is a programmable multi-gas monitor designed to provide continuous exposure monitoring of carbon dioxide, toxic gases, oxygen and combustible gases (VOCs) for workers in potentially hazardous environments. Readings were obtained over a range of one hour, repeated three times, and then the average was recorded.

Noise levels at all sampling locations were measured using a noise meter (CEM Digital Noise Meter – Model GT – 805). The sensitivity ranges from 30 to 130 dB, with air frequencies between 31.5 Hz and 8 kHz. The sensor of the noise meter was directed towards the source of the noise and the average readings over a period of one hour were taken to be the noise-level at each point.^25^

### Distribution map development

The obtained readings with their respective coordinates were computed into ArcGIS 10 software and distribution maps were obtained using pre-defined ranges for the respective air quality parameters. The maps were presented as colored gradients representing the distribution pattern of the respective ranges of the parameters.

### Statistical analysis

The obtained readings were processed using SPSS Version 20 Software (IBM) and values were presented as means. The respective readings were also subjected to statistical analysis and significant means at P<0.05 were separated using independent samples T-Test.

## Results

The air sampling operation indicated varying air quality parameters for residential areas (*Supplemental Material 1*) and TSLs (transport sector locations include busy roads, trunk A roads (dual carriage) and busy bus stops) (*Supplemental Material 2*).

### Residential air quality

**Figure 2:**
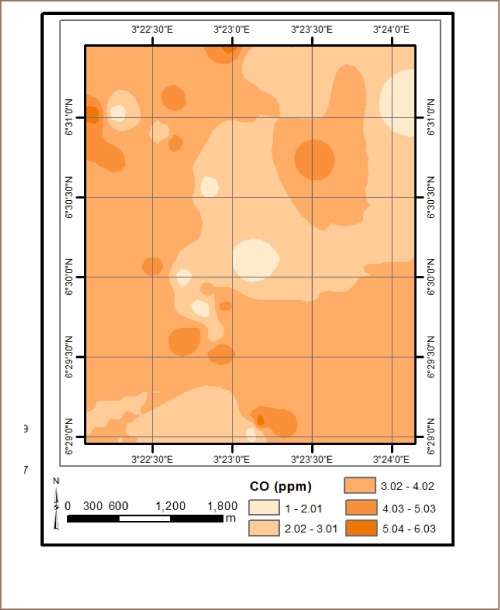
Carbon monoxide (ppm) distribution around residential areas and transport sector locations

**Figure 3:**
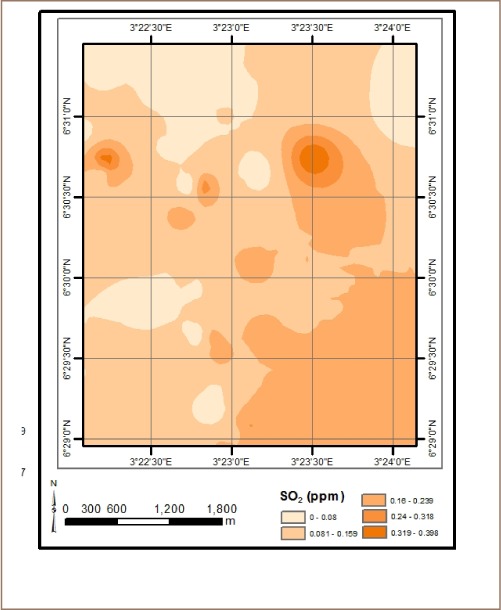
Sulphur dioxide (ppm) distribution around residential areas and transport sector locations

**Figure 4:**
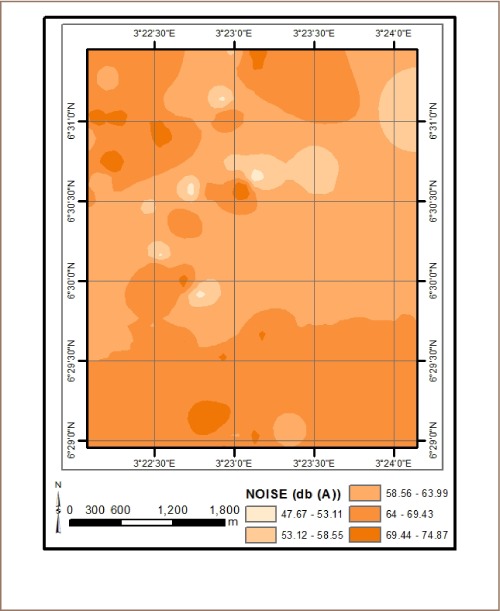
Noise distribution (db (A)) around residential areas and transport sector locations

**Figure 5:**
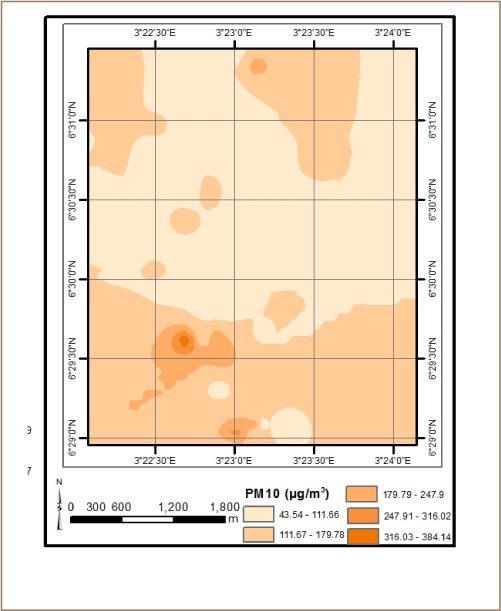
PM_10_ (μg/m^3^) distribution around residential areas and transport sector locations

**Figure 6:**
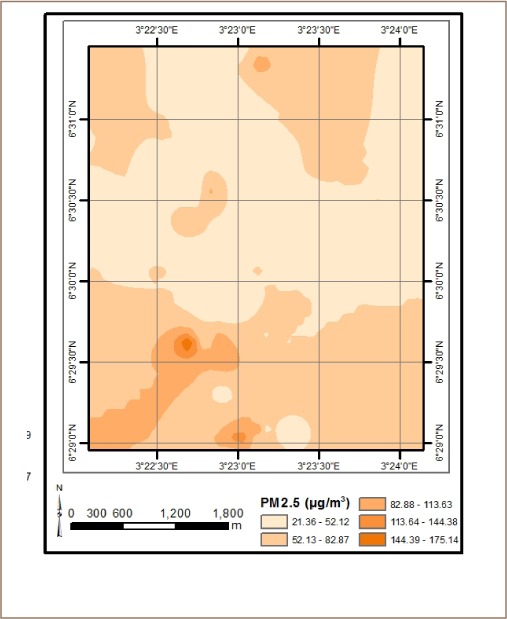
PM_2.5_ (μg/m^3^) distribution around residential areas and transport sector locations

**Figure 7:**
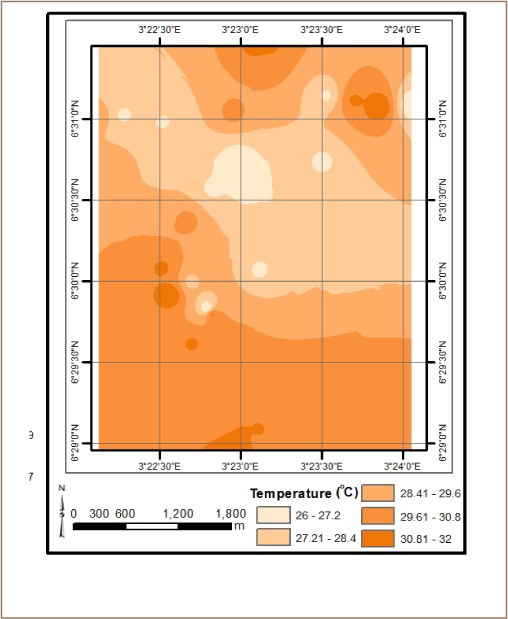
Temperature (°C) distribution around residential areas and transport sector locations

**Figure 8:**
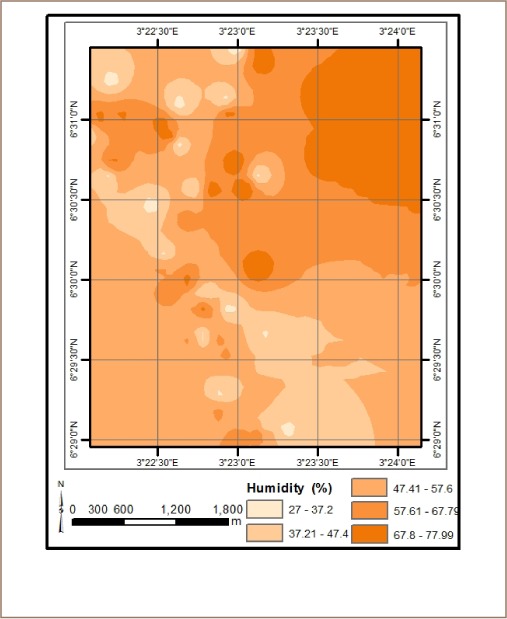
Humidity (%) distribution around residential areas and transport sector locations

The results from *in situ* assessment of air quality around residential areas in Lagos indicated that H_2_S, VOCs, NO_2_ and NH_3_ were low, and values were below the detection limit. However, other parameters such as CO, SO_2_, noise, PM, temperature and humidity were within measurable levels. With respect to CO levels, detected values ranged from 1.0 ppm at various locations to 6.0 ppm at the busy Ebutemeta Street, outside of the Makoko slum (*Figure 2*). The SO_2_ levels were low and ranged from 0.0 ppm in almost half of the samples to 0.2 ppm at the Ebutemeta Street location (*Figure 3*). Noise levels around the residential areas ranged from 47.7 dB(A) at the houses around Mbonu Ojike Close at the University Campus, to 70.1 dB(A) at the busy Ebutemeta Street (*Figure 4*). The PM_10_ values in the air around the residential areas ranged from 43.3 μg/m^3^ at Majoro Street, off Onike to 159.7 μg/m^3^ in the residential area around Igbobisare Street, WAEC bus stop (*Figure 5*). The PM_2.5_ values ranged from 20.3 μg/m^3^ around Abudu Street, Abuleoja, to 69.0 μg/m^3^ around Igbobisare Street, WAEC bus stop (*Figure 6*). Air temperatures around the residential areas ranged from 27.2 to 36.7°C (*Figure 7*), and humidity values ranged from 57.6 to 92.3% around the densely vegetated Mbonu Ojike Close (*Figure 8*).

### Transport sector location air quality

The results of the air quality parameters around the TSLs showed a wide range of values which depend on the typical vehicular density at each location. The CO levels ranged from 1.0 to 7.7 ppm at the University Junction (*Figure 2*), and 0.0 to 0.4 ppm for SO_2_ (Distance Learning Institute (DLI) Junction, University of Lagos (UNILAG)) (*Figure 3*). The VOC levels in the TSLs were very low and ranged from 0.0 to 0.1 ppm. NO_2_, NH_3_, H_2_S, were not detected at all transport locations, and noise levels ranged from 53.1 dB(A) (Zoological Garden - control) to 76.3 dB(A) (Barracks Bus Stop) (*Figure 4*). With respect to particulate matter, detected PM_10_ levels ranged from 60.7 μg/m^3^ at the Zoological Garden (control location) to 404.0 μg/m^3^ (at Adekunle) (*Figure 5*). PM_2.5_ levels were lowest at the Zoological Garden (control location) and ranged from 32.3 to 184.0 μg/m^3^ (*Figure 6*). The temperature around TSLs ranged from 26.3 to 32.6°C (*Figure 7*). The lowest temperatures were recorded at the Zoological Garden (control location). Humidity ranged from 60 to 78% (*Figure 8*), with the high humidity of the control location (Zoological Garden) likely due to the dense vegetation around the area.

### Comparison of air quality around residential and transport sector locations

The results comparing the overall air quality parameters around residential areas and TSLs are presented in Table 1. The results of the T-test comparison of means are also shown. CO levels at residential areas and TSL readings indicated no significant differences (P >0.05). Overall mean SO_2_ levels were significantly higher (P <0.005) for the TSLs than residential areas, with values of 0.14 ± 0.09 ppm and 0.06 ± 0.07 ppm, respectively. The mean noise levels for the TSLs and residential areas were 67.7 ± 6.0 dB(A) and 58.2 ± 6.8 dB(A), respectively, indicating a significant difference (P < 0.05). The mean PM_10_ levels for the TSLs and residential areas were 144.1 ± 76.6 μg/m^3^ and 73.4 ± 27.5 μg/m^3^, respectively, with significantly higher values for the former (P <0.001). The overall mean PM_2.5_ levels for the TSLs were also significantly higher (P <0.001) than the levels around residential areas, with values of 69.6 ± 35.1 μg/m^3^ and 34.9 ± 12.1 μg/m^3^, respectively. The overall mean VOC level for the TSLs was 0.023 ± 0.04 ppm and was below the detection limit for households, indicating a significant difference (P < 0.05). Temperatures around residential areas were significantly lower (29.2 ± 2.0°C) than those measured around TSLs (33.3 ± 2.2°C). There was no significant difference in the overall mean values of humidity for the residential areas and TSLs.

## Discussion

The present study examined the levels and spatial distribution of key air quality parameters within residential areas and TSLs (primarily bus stops) in Lagos, Nigeria. The assessed parameters, including carbon monoxide (CO), sulphur dioxide (SO_2_), noise, particulate matter (PM_10_ and PM_2.5_), hydrogen sulphide (H_2_S), ammonia (NH_3_), nitrogen oxide (NO_2_), volatile organic carbon (VOCs), temperature and humidity have been used as indicators of air quality in previous studies.^15^,^29^

Among the key parameters assessed was CO, which despite its colorless and odorless properties is highly toxic, with the ability to combine with hemoglobin to produce carboxyhemoglobin. When inhaled, this compound takes up the space in hemoglobin that normally carries oxygen, but is ineffective for delivering oxygen to bodily tissues.^30^ Concentrations as low as 667 ppm may cause up to 50% of the body's hemoglobin to convert to carboxyhemoglobin.^31^ The fact that these levels were not observed in the sampling locations in this study is reassuring. However, a more important consideration is the effect of chronic exposures to low doses of this pollutant in cases of occupational exposure or high doses in accidental exposures in homes and workplaces. A level of 50% carboxyhemoglobin may result in seizure, coma, and death. These effects can take place over short durations, as carbon monoxide absorption is cumulative, with a half-life of about 5 hours in fresh air.^32^

The levels of CO in the study area were below the Nigerian FMEnv regulatory limit of 10 ppm.^28^ The fact that CO levels did not differ significantly between residential areas and TSLs can be linked to the persistent use of generating sets for electricity in residential areas. This, together with emissions from cooking, contributes to CO emission levels which did not differ significantly from levels at TSLs.

Suspended particulate matter can remain in the air for long periods of time and is a key component of air pollution and smog. Particulate matter affects the environment as it contributes to greenhouse gases.^26^ It affects human health as PM and can easily reach the deepest parts of the lungs, leading to respiratory ailments.^26^ The levels of PM_10_ in the study area were significantly higher in TSLs and ranged between 60.7 and 404.0 μg/m^3^ at the TSLs and between 43.3 and 159.61 μg/m^3^ in residential areas. This demonstrates that the particulate levels present in the atmosphere were higher than the USEPA regulatory limits of 150 μg/m^3^ and in some cases exceeded the Nigerian FMEnv limit of 250 μg/m^3^, especially around some TSLs like the Jebba and Adekunle bus-stops.^26^,^28^ In addition, the levels of PM_2.5_ in the study area for TSLs ranged between 32.3 to 184.0 μg/m^3^ and 20.3 to 69.0 μg/m^3^ in residential areas. This shows that the particulates present in the atmosphere exceeded the USEPA limit of 35 μg/m^3^ in some residential locations, but well below the FMEnv limit of 250 μg/m^3^.^26^,^28^ High values of PM_2.5_ in TSLs may be attributed to vehicular exhaust due to the relatively high traffic along major roads and bus stops. The high values obtained in some residential areas can be attributed to exhaust fumes from diesel and petrol generator sets which are commonly used in household locations to make up for shortages due to frequent power outages from the national grid. The size of particulate matter plays an important role in environmental health risk. PM is categorized by aerodynamic diameter. Particles below 10 μm in diameter are classified as thoracic particles (PM_10_), particles below 2.5 μm in diameter as fine particles (PM_2.5_), and particles with a diameter < 0.1 μm as ultrafine particles.^33^ Particulate matter is hazardous to human health due to the adsorption of many harmful contaminants on its surface including heavy metals (lead, cadmium, mercury and the other) and organic compounds (polycyclic aromatic hydrocarbons, polychlorinated biphenyls, dioxin and furans). Ambient fine particulate pollution has been associated with increased risk of cardiovascular diseases.^34^

The levels of SO_2_ recorded in the study area for TSLs ranged between 0.0 to 0.4 ppm and between 0.0 and 0.17 ppm around residential locations. Locations around the busy TSLs such as the Yaba bus-stop, Kano and Abeokuta Street had levels that were above the USEPA regulatory limit of 0.1 ppm.^26^ A study in China found that air pollutants such as SO_2_ may contribute to an increased risk for lung cancer mortality.35 Inhalation of SO_2_ is associated with increased respiratory symptoms and disease, difficulty in breathing, and premature death.^36^ In addition, the concentration of SO_2_ in the atmosphere can influence habitat suitability for plant communities, as well as animal life. Their emissions are also a precursor to acid rain and atmospheric particulates.^37^

The levels of VOCs recorded in the study area for TSLs ranged between 0.0 to 0.1 ppm and were below the detection limit around residential locations. The atmospheric oxidation of VOCs can produce secondary pollutants such as ground level ozone or peroxy acetyl nitrate.^38^ VOCs are typically released into the atmosphere from anthropogenic actives and biogenic sources. Anthropogenic sources are dominated by the combustion of fossil fuels, and this may have led to the detection of these gases in some TSLs. Biogenic sources include releases from plant foliage, and the microbial decomposition of organic substances, fresh and marine waters, soils, sediments and geological resources of hydrocarbons are additional natural sources of these compounds.^39^

## Conclusions

Air quality, especially with respect to PM concentrations in the study area around TSLs was poor, as this pollutant was above the limits set by the USEPA and FMEnv at a number of sampling locations. Although values of some other parameters were relatively high, they did not exceed set limits. Most assessed parameters were also found to be significantly higher than those measured around residential areas, except for CO. The high levels of air quality parameters detected in some of the sampling locations in the present study are a greater consideration than the extent to which they exceeded set limits, as chronic exposure to minute concentrations plays a greater role in toxicology than acute exposures. The effects of poor air quality on public health and urban wildlife is a cause for concern in view of the increasing incidence of cancer diagnosis in Nigeria and the existing pressure on wildlife. In keeping with the current trend globally, developing countries such as Nigeria must embrace cleaner technology and continually reduce the levels of fossil fuels used in automobiles and internal combustion systems by adopting more efficient technologies. This should be enforced by legislature with feasible timelines which take into account public health and economic factors.

## References

[1] Katulski RJ, Namiessnik J, Sadowski J, Stefannski J, Wardencki W, Khallaf M (2011). Monitoring of gaseous air pollution [Internet]. The impact of air pollution on healthy economy, environment and agricultural sources.

[2] (2014). 7 million deaths annually linked to air pollution [Internet].

[3] (c2018). WHO global urban ambient air pollution database (update 2016) [Internet].

[4] Mohan D, Thiyagarajan D, Murthy PB (2013). Toxicity of exhaust nanoparticles. Afr J Pharm Pharmacol [Internet].

[5] Bernstein J, Alexis N, Barnes C, Bernstein IL, Bernstein JA, Nel A, Peden D, Diaz-Sanchez D, Tarlo SM, Williams PB (2004). Health effects of air pollution. J Allergy Clin Immunol [Internet].

[6] (2008). Care for your air: a guide to indoor air quality [Internet].

[7] Saura M, Zaragoza C, Bao C, Herranz B, Rodriguez-Puyol M, Lowenstein CJ (2006). Stat3 mediates interleukin-6 [correction of interelukin-6] inhibition of human endothelial nitric-oxide synthase expression. J Biol Chem [Internet].

[8] Wargo J, Wargo L, Alderman N (2006). The harmful effects of vehicle exhaust: a case for policy change [Internet].

[9] Wegesser TC, Last JA (2009). Mouse lung inflammation after instillation of particulate matter collected from a working dairy barn. Toxicol Appl Pharmacol [Internet].

[10] Fortoul TI, Rojas-Lemus M, Rodriguez-Lara V, Cano-Gutierrez G, Gonzalez-Villalva A, Ustarroz-Cano M, Garcia-Pelaez I, Lopez-Valdez N, Falcon-Rodriguez CI, Silva-Martinez J, Gonzalez-Rendon ES, Montano LF, Cano-Gutierrez B, Bizarro-Nevares P, Barenque CL, Khallaf M (2011). Air pollution and its effects in the respiratory system [Internet]. The impact of air pollution on healthy economy, environment and agricultural sources.

[11] Emmerechts J, Jacobs L, Hoylaerts MF, Khallaf M (2011). Air pollution and cardiovascular disease [Internet]. The impact of air pollution on healthy economy, environment and agricultural sources.

[12] Akpan KV, Sogbanmu TO, Otitoloju AA (2012). Influence of volatile organic solvents' inhalation on activity quotient and biochemical indices of Mus musculus. J Environ Occup Sci [Internet].

[13] Bostrom CE, Gerde P, Hanberg A, Jernstrom B, Johansson C, Kyrklund T, Rannug A, Tornqvist M, Victorin K, Westerholm R (2002). Cancer risk assessment, indicators, and guidelines for polycyclic aromatic hydrocarbons in the ambient air. Environ Health Perspect [Internet].

[14] Raub JA, Mathieu-Nolf M, Hampson NB, Thom SR (2000). Carbon monoxide poisoning--a public health perspective. Toxicology [Internet].

[15] Ramanathan V, Feng Y (2009). Air pollution, greenhouse gases, and climate change: global and regional perspectives. Atmos Environ [Internet].

[16] Flanner MG, Zender CS, Randerson JT, Rasch PJ (2007). Present-day climate forcing and response from black carbon in snow. J Geophys Res [Internet].

[17] Pounds JA, Bustamante MR, Coloma LA, Consuegra JA, Fogden MP, Foster PN, La Marca E, Masters KL, Merino-Viteri A, Puschendorf R, Ron SR, Sanchez-Azofeifa GA, Still CJ, Young BE (2006). Widespread amphibian extinctions from epidemic disease driven by global warming. Nature [Internet].

[18] Mannucci PM, Franchini M (2017). Health effects of ambient air pollution in developing countries. Int J Environ Res Public Health [Internet].

[19] (c2018). Ambient (outdoor) air quality and health [Internet].

[20] Harfenist E (2016). Air pollution is choking cities in low-income countries. Vocativ [Internet].

[21] Ibem EO (2011). Challenges of disaster vulnerability reduction in Lagos Megacity Area, Nigeria. Disaster Prev Manag [Internet].

[22] (2009). Global health risks: mortality and burden of disease attributable to selected major risks [Internet].

[23] John KS, Feyisayo K (2013). Air pollution by carbon monoxide (CO) poisonous gas in Lagos Area Southwestern Nigeria. Atmos Clim Sci [Internet].

[24] Njoku KL, Rumide TJ, Akinola MO, Adesuyi AA, Jolaoso AO (2016). Ambient air quality monitoring in Metropolitan City Of Lagos, Nigeria. J Appl Sci Environ Manag [Internet].

[25] Aery NC (2010). Manual of environmental analysis.

[26] (1999). National emission standards for hazardous air pollutants for source categories: Portland cement manufacturing industry. Federal Register [Internet].

[27] (c2006). WHO air quality guidelines for particulate matter, ozone, nitrogen dioxide and sulfur dioxide [Internet].

[28] (1995). Nigerian ambient air quality standard.

[29] Ameh JA, Tor-Anyiin TA, Eneji IS (2015). Assessment of some gases emissions in traffic areas in Makurdi metropolis, Benue State, Nigeria. OJAP [Internet].

[30] Rim-Rukeh A (2014). An assessment of the contribution of municipal solid waste dump site fire to atmospheric pollution. OJAP [Internet].

[31] Tikuisis P, Kane DM, McLellan TM, Buick F, Fairburn M (1992). Rate of formation of carboxyhaemoglobin in exercising humans exposed to carbon monoxide. J Appl Physiol.

[32] Haradhan KM (2014). Chinese Sulphur Dioxide Emissions and Local Environment Pollution. International Journal of Scientific Research in Knowledge.

[33] Brook RD, Franklin B, Cascio W, Hong Y, Howard G, Lipsett M, Luepker R, Mittleman M, Samet J, Smith SC, Tager I (2004). Air pollution and cardiovascular disease: a statement for healthcare professionals from the Expert Panel on Population and Prevention Science of the American Heart Association. Circulation [Internet].

[34] Pope CA, Muhlestein JB, May HT, Renlund DG, Anderson JL, Horne BD (2006). Ischemic heart disease events triggered by short-term exposure to fine particulate air pollution. Circulation [Internet].

[35] Cao J, Yang C, Li J, Chen R, Chen B, Gu D, Kan H (2011). Association between long-term exposure to outdoor air pollution and mortality in China: a cohort study. J Hazard Mater [Internet].

[36] Jack EP, Richard DS (1970). Absorption and Elimination of Carbon Monoxide by Inactive Young Men. Arch Environ Occup Health.

[37] Don-Pedro KN (2009). Man and the environmental crises.

[38] Bakeas EB, Siskos PA (2002). Volatile hydrocarbons in the atmosphere of Athens, Greece. Environ Sci Pollut Res Int [Internet].

[39] Guenther A, Hewitt CN, Erickson D, Fall R, Geron C, Graedel T, Harley P, Klinger L, Lerdau M, Mckay WA, Pierce T, Scholes B, Steinbrecher R, Tallamraju R, Taylor J, Zimmerman P (1995). A global model of volatile organic compound emissions. J Geophys Res [Internet].

