# Supplementary material for: Air Pollution Monitoring Around Residential and Transportation Sector Locations in Lagos Mainland
Source: J Health Pollut. 2018 Aug 21;8(19):180903. doi: 10.5696/2156-9614-8.19.180903 (PMC6257165; doi:10.5696/2156-9614-8.19.180903)
Supplement: Supplementary file 1 [file Obanya_Supplemental_Material_1.pdf]

Supplemental Material 1

Air quality around households in the study area

S/N	CO (ppm)	SO_2 (ppm)	VOCs (ppm)	NO_2 (ppm)	NH_3 (ppm)	H_2S (ppm)	Noise (dB)	$PM_{2.5}$ (μg/m^3)	PM_{10} (μg/m^3)	Temp. (^{o}C)	Humidity (%)
1	2.0	0.0	ND	ND	ND	ND	47.7	43.7	91.0	27.2	92 .3
2	4.0	0.0	ND	ND	ND	ND	57.0	28.7	51.7	30.1	83.1
3	4.0	0.0	ND	ND	ND	ND	50.9	20.3	47.3	34.1	71 .4
4	5.0	0.0	ND	ND	ND	ND	65.8	26.3	55.7	32.3	73.3
5	2.0	0.0	ND	ND	ND	ND	61.1	23.3	48.0	32.3	78.6
6	3.0	0.1	ND	ND	ND	ND	62.6	69.0	159.7	33.3	71.3
7	3.0	0.1	ND	ND	ND	ND	65.9	33.0	66.0	34.8	65 .5
8	2.0	0.1	ND	ND	ND	ND	67.7	27.0	53.7	34.1	66.1
9	3.0	0.2	ND	ND	ND	ND	58.5	30.3	64.7	33.5	57.6
10	1.0	0.1	ND	ND	ND	ND	65.3	32.0	72.7	35.4	60.3
11	6.0	0.2	ND	ND	ND	ND	70.1	51.3	101.3	36.4	63.6
12	5.0	0.1	ND	ND	ND	ND	60.6	26.0	52.7	31.1	61.3
13	4.0	0.0	ND	ND	ND	ND	56.8	43.3	93.0	34.4	67.7
14	5.0	0.1	ND	ND	ND	ND	59.7	48.0	102.0	34.6	65.8
15	4.0	0.1	ND	ND	ND	ND	57.1	35.7	76.0	33.6	70.3
16	5.0	0.1	ND	ND	ND	ND	51.8	34.0	77.7	35.3	65.3
17	2.0	0.0	ND	ND	ND	ND	49.2	45.7	93.0	36.7	60.4
18	2.0	0.0	ND	ND	ND	ND	61.9	42.3	86.7	31.0	61.2
19	2.0	0.1	ND	ND	ND	ND	53.7	25.0	50.7	36.5	61.3
20	1.0	0.0	ND	ND	ND	ND	49.1	25.3	55.3	35.4	63.3
21	1.0	0.0	ND	ND	ND	ND	49.0	22.3	43.3	35.1	63.4

Abbreviation: ND, below detection limit. Values are mean ± standard deviation of 3 replicates.
1- Mbonu Ojike Close; 2-Oluwole Street, Pako; 3- Abudu Street, Abuleoja; 4- Ibikunle Street, University Junction; 5- Yabatech College quarters; 6- Igbobisare Street, WAEC Bus Stop; 7- Glover Street, Post office; 8- Jebba Street; 9- Osholanke Street; 10- Oloto Street; 11- Ebutemeta Street, off Makoko; 12-Adebosi Street, Police Corporative; 13- Okesuna Street, Jibowu; 14- Biney Street, Yaba; 15- Ireti Street, Sabo; 16- Borno Way, Spenser; 17- Moleye Street, Alagomeji; 18- Simpson Street, Adekunlebusstop; 19- Queen Street, Casino; 20- St. Agnes Street, Barracks; 21- Majoro Street, off Onike
