# Supplementary material for: Air Pollution Monitoring Around Residential and Transportation Sector Locations in Lagos Mainland
Source: J Health Pollut. 2018 Aug 21;8(19):180903. doi: 10.5696/2156-9614-8.19.180903 (PMC6257165; doi:10.5696/2156-9614-8.19.180903)
Supplement: Supplementary file 2 [file Obanya_Supplemental_material_2.pdf]

Supplemental Material 2

Air quality around transport sector locations in the study area

S/N	CO (ppm)	SO2 (ppm)	VOCs (ppm)	NO_2 (ppm)	NH_3 (ppm)	H_2S (ppm)	Noise (dB)	PM_{10} ($\mu g/m^3$)	$PM_{2.5}$ ($\mu g/m^3$)	Temp (^oC)	Humidity (%)
1	5.0	0.1	0.1	ND	ND	ND	68.6	155.0	74.3	27.2	71
2	5.7	0.1	0.0	ND	ND	ND	65.2	112.3	56.0	31.1	74
3	3.3	0.1	0.0	ND	ND	ND	65.2	114.0	57.3	32.4	70
4	5.0	0.4	0.0	ND	ND	ND	55.7	123.0	56.3	27.6	70
5	4.7	0.1	0.0	ND	ND	ND	70.2	220.7	100.0	30.2	74
6	3.0	0.1	0.0	ND	ND	ND	72.1	119.0	57.0	32.1	72
7	3.0	0.1	0.1	ND	ND	ND	70.1	107.0	51.0	30.2	72
8	7.7	0.1	0.0	ND	ND	ND	72.5	108.0	57.0	28.5	76
9	4.7	0.1	0.1	ND	ND	ND	70.3	135.0	59.3	27.4	74
10	3.3	0.1	0.0	ND	ND	ND	70.5	123.0	65.3	27.6	70
11	4.0	0.1	0.1	ND	ND	ND	71.9	162.0	74.0	29.4	72
12	4.7	0.4	0.0	ND	ND	ND	75.9	127.0	58.0	29.4	74
13	4.3	0.2	0.0	ND	ND	ND	69.7	132.0	63.3	30.1	62
14	3.3	0.1	0.0	ND	ND	ND	69.9	133.0	58.0	31.5	60
15	3.0	0.0	0.0	ND	ND	ND	69.7	102.3	54.3	32.6	61
16	3.0	0.1	0.0	ND	ND	ND	70.0	404.0	184.0	31.2	60
17	2.7	0.0	0.0	ND	ND	ND	75.0	140.0	70.0	30.7	60
18	4.7	0.2	0.0	ND	ND	ND	72.2	115.3	61.0	32.2	61
19	4.0	0.1	0.0	ND	ND	ND	62.1	290.0	140.7	31.6	60
20	4.0	0.3	0.0	ND	ND	ND	69.6	294.0	147.0	31.3	62
21	3.0	0.2	0.1	ND	ND	ND	70.8	83.0	44.7	30.3	64
22	5.0	0.2	0.1	ND	ND	ND	70.8	226.0	103.3	30.2	60
23	2.0	0.2	0.1	ND	ND	ND	61.7	222.0	101.0	30.1	61
24	1.0	0.1	0.0	ND	ND	ND	61.1	83.0	44.0	31.5	60
25	1.0	0.1	0.0	ND	ND	ND	60.0	74.3	37.0	26.2	78
26	1.0	0.1	0.0	ND	ND	ND	73.2	72.0	33.0	28.3	74
27	1.0	0.3	0.0	ND	ND	ND	67.3	171.0	90.0	27.1	76
28	2.0	0.1	0.0	ND	ND	ND	76.3	69.0	33.7	27.6	76
29	2.0	0.1	0.0	ND	ND	ND	56.6	77.0	41.7	26.3	76
30	1.0	0.2	0.0	ND	ND	ND	60.6	111.3	53.0	27.2	78
31	1.0	0.0	0.0	ND	ND	ND	53.1	60.7	32.3	26.2	78

Abbreviation: ND, below detection limit. Values are mean ± standard deviation of 3 replicates.

1- New Hall, University of Lagos; 2- CITS, University of Lagos; 3- Cab Station, University of Lagos; 4- DLI Junction, University of Lagos ; 5- St. Finbars; 6- Pako; 7- Abuleoja Junction; 8- University Junction; 9- Yabatech; WAEC Bust Stop; 10- Jibowu; 11- Yaba; 12- Sabo Bus stop; 13- Spenser; 15- Alagpmeji II; 16- Adekunle; 17- Post Office; 18- Oyingbo; 19- Jebba 1; 20- Jebba II; 21-Kano Street; 22- Adekunle 2; 23- Makoko; 24- Police Corporative; 25- Casino; 26- Alagomeji; 27- Barracks; 28- Onike Round About; 29- Araromi; 30- University Gate; 31- Zoological Garden (Control)
